# Aging and radiation: bad companions

**DOI:** 10.1111/acel.12306

**Published:** 2015-02-02

**Authors:** Laia Hernández, Mariona Terradas, Jordi Camps, Marta Martín, Laura Tusell, Anna Genescà

**Affiliations:** 1 Department of Cell Biology, Physiology and Immunology, Universitat Autònoma de Barcelona08193, Bellaterra, Spain; 2 Gastrointestinal and Pancreatic Oncology Group, Hospital Clínic, Centro de Investigación Biomédica en Red de Enfermedades Hepáticas y Digestivas (CIBERehd), Institut D'Investigacions Biomèdiques August Pi i Sunyer (IDIBAPS)08036, Barcelona, Spain

**Keywords:** chromatin organization, DNA repair, nuclear envelope, oxidative stress, replicative senescence, radiosensitivity

## Abstract

Aging involves a deterioration of cell functions and changes that may predispose the cell to undergo an oncogenic transformation. The carcinogenic risks following radiation exposure rise with age among adults. Increasing inflammatory response, loss of oxidant/antioxidant equilibrium, ongoing telomere attrition, decline in the DNA damage response efficiency, and deleterious nuclear organization are age-related cellular changes that trigger a serious threat to genomic integrity. In this review, we discuss the mechanistic interplay between all these factors, providing an integrated view of how they contribute to the observed age-related increase in radiation sensitivity. As life expectancy increases and so it does the medical intervention, it is important to highlight the benefits of radiation protection in the elderly. Thus, a deep understanding of the mechanistic processes confining the threat of aging-related radiosensitivity is currently of foremost relevance.

## Introduction

Age at the time of radiation exposure is one of the main factors involved in radiation-induced cancer. Individuals exposed at early ages are the most radiosensitive as the primary damage has a longer latent phase to outbreak into cancer. After that, sensitivity to radiation decreases until maturity, but it increases again at older ages. Epidemiological evidence for such age-dependent radiosensitivity variation has been reported in different studies. In the Life Span Study cohort of the Japanese atomic bomb survivors, the excess relative risks (ERRs) for radiation-induced cancers as a function of age at exposure were examined (Shuryak *et al*., 2010). As expected, the ERR for cancer induction was higher during childhood and decreased progressively at exposure ages of 30–40. Surprisingly, the ERR of developing solid tumors ramped up again for exposure ages higher than 40 years old (Shuryak *et al*., 2010). Richardson and Wing found a similar tendency among the Oak Ridge Y-12 uranium processing plant workers (Richardson & Wing, 1999). They observed that the radiation doses received after the age of 45 showed a stronger association with cancer mortality than those received at younger ages. All these findings suggest that the radiation sensitivity, measured in terms of carcinogenic events, increases with age among adults after age of 40–45. It has been suggested that this bimodal distribution reflects that radiation risks after exposure at early ages are related to initiation of malignant processes, whereas radiation risks after exposure at later ages are mostly associated with the promotion of pre-existing premalignant cells (Shuryak *et al*., 2010). Not only radiosensitivity, but also the percentage of individuals undergoing medical imaging procedures becomes significantly greater with age, and so does the cumulative effective dose from these procedures (Fazel *et al*., 2009). Finally, life expectancy has remarkably increased in the last decades and thus, radiation-induced tumors at older ages have more time to develop and progress.

Therefore, understanding the age-related changes that may compromise the population after radiation exposure becomes increasingly relevant. In this review, we will focus on four cellular processes: oxidative stress, telomere attrition, DNA repair, and inflammatory response, and we will provide an integrated view of how these four mechanisms may contribute to the relationship between aging and radiosensitivity.

## Oxidative stress arising from aging and radiation

### Lessons from the free radical aging theory

Reactive oxygen species (ROS) can be endogenously generated by the normal cellular metabolism or exogenously originated by exposure to radiation and chemical compounds (Valko *et al*., 2007). ROS may form compounds, such as hydroxyl radicals or hydrogen peroxide, which could initiate harmful chemical reactions in cells. High levels of ROS can cause damage to macromolecules, such as lipids, nucleic acids, and proteins. Lipid peroxidation is one of the early steps of this damaging process, followed by oxidation of nitrogenated bases. Damage to the DNA, mainly strand breaks and DNA cross-linking, is a source of mutations in the cell genome. In addition, practically, every amino acid in a protein can be oxidized by ROS. The harmful effects of ROS are termed as oxidative stress. However, the damage that ROS can induce to the cell not only depends on their concentration but also on the equilibrium between ROS and the antioxidant species. Oxidative stress is generated when there is a loss of pro-oxidant–antioxidant equilibrium, thus altering and damaging many intracellular molecules, including DNA, RNA, lipids, and proteins (Veskoukis *et al*., 2012). Oxidative stress is important from a biomedical perspective because it is linked to a wide variety of human diseases, such as neurodegenerative and inflammatory diseases, as well as cancer. ROS can promote many aspects of tumor development and progression at different levels: cellular proliferation, evasion of apoptosis, tissue metastasis and invasion, as well as angiogenesis (reviewed in Sosa *et al*., 2013).

As ROS constitute a persistent source of DNA damage, they were assumed to contribute to the age-related deterioration of functions in the organism. The oldest theory that relates aging with ROS was proposed more than 50 years ago and postulates that the accumulation of ROS and the subsequent oxidative damage along the lifespan of aerobic organisms triggers the aging process (Harman, 1956, 1992). Recently, this theory has been severely criticized by some authors, who claim that the correlation between oxidative damage and the aged phenotype, even if it exists, does not imply causation (Buffenstein *et al*., 2008; Pérez *et al*., 2009; Lapointe & Hekimi, 2010). While entering in this debate is beyond the scope of this article, some points of the theory are relevant to this discussion. First, contributing to aging or not, ROS accumulation was found to be higher in aged cells in comparison to their young counterparts (Ku *et al*., 1993). Secondly, in addition to the increased ROS, impaired antioxidative enzymatic activities with aging have also been described. Antioxidant enzymes such as superoxide dismutase, catalase and glutathione peroxidase showed a decline in their activities and/or gene expression with age (Andersen *et al*., 1997; Inal *et al*., 2001). Nowadays, the free radical aging theory seems to evolve toward a more conservative position, which states that there is an increasing imbalance between the number of oxidant and antioxidant species with age (Junqueira *et al*., 2004). This imbalance may lead to age-dependent oxidative stress that compromises both cellular structures and homeostasis (Kregel & Zhang, 2007; Liu & Xu, 2011).

### Ionizing radiation worsens the already unbalanced oxidant–antioxidant status in aging cells

**Figure 1:**
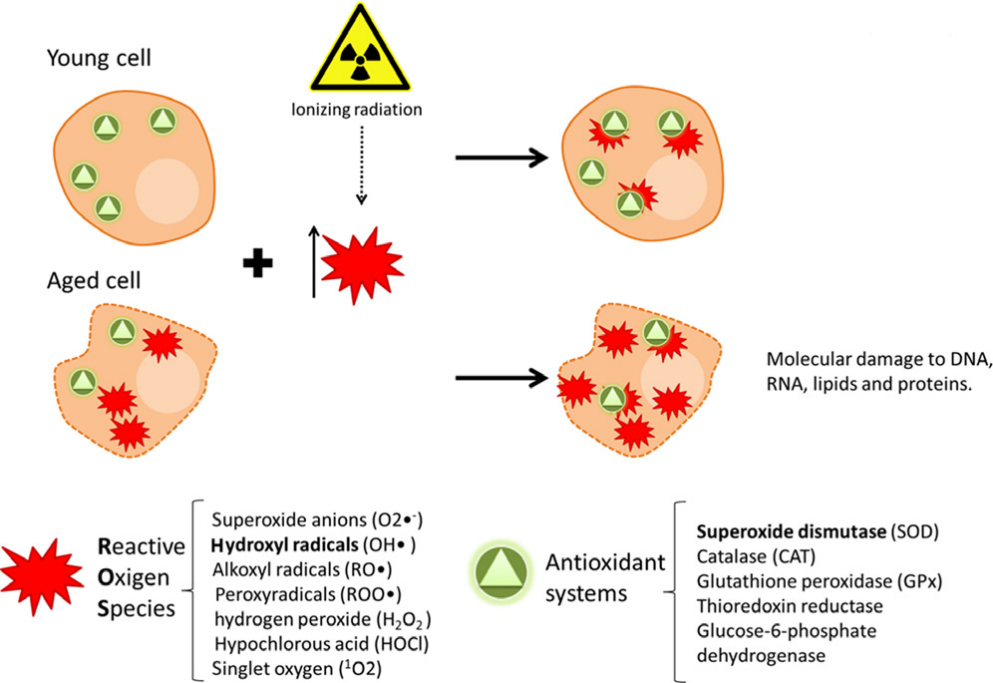
Progressive loss of the pro-oxidant/antioxidant equilibrium with age and the synergic effect that ionizing radiation has on this process. Reactive oxygen species (ROS) production is greater in aging cells in comparison to their young counterparts; in addition, the antioxidant system is also compromised in these cells. This scenario leads to an increase in the oxidative stress. When adding ionizing radiation into this equation, the system becomes oversaturated, resulting in increased amounts of cell damage in aging cells. Red stars represent the ROS and the green triangles the antioxidant systems/enzymes.

Ionizing radiation can have various effects at the cellular level. When radiation is absorbed by the cell, damage can occur either by direct or indirect action. In a direct action, a secondary electron, which results from photon absorption, interacts with and is absorbed by a biomolecule, such as DNA, RNA, or proteins. In an indirect action, ionizing radiation impairs or damages cells by creating free radicals (Riley, 1994). Radiation results in the high local production of ROS attributable to chemical interactions between high-energy electrons, photons, and the molecular targets of oxygen and water within cells. Radiation can also produce ROS through signaling processes that evolve in their release from mitochondria. It has been proposed that intrinsic sensitivity of cells to radiation is dependent on the subsequent ROS generation as they can induce mutations in the DNA (Seong *et al*., 2010). Irradiating aged cells that already contain an increased amount of ROS would unequivocally contribute to overload the antioxidant systems responsible for eliminating the excess of oxygen metabolites (Fig.1). Importantly, in an attempt to estimate the effect of aging on radiation-dependent ROS generation, Kasapovic *et al*. (2009) provided evidence that older women had a decreased antioxidant capacity and subsequent higher oxidative damage. The activity of the different antioxidant enzymes and concentration of lipid hydroperoxides were measured in blood cells of two groups of breast cancer patients with different ages, to estimate their capacity to eliminate H_2_O_2_ formed after radiotherapy. A decreased antioxidant capacity and an increased lipid peroxidation were observed in the older patient group. The authors concluded that radiotherapy promotes further oxidative shift, which in turn potentiates the already existing chronic oxidative stress linked to breast cancer and aging, resulting in a further increase of mutagenic potential. Similarly, antioxidant protection against curative and palliative doses of ionizing radiation in human blood was reported to decrease with aging (Kasapovic *et al*., 2009). Thus, radiation may cause a major impact in the elderly, whose aging cells already naturally showed an impaired redox system. In line with this, *in vivo* experimental studies on animal models have shown that adding antioxidant supplements to the diet can minimize the effects of ionizing radiation later on (Weiss & Landauer, 2003). V*ice versa,* approaches were achieved with knocked-down cytoglobin (a vertebrate globin that scavenges ROS), which made glioma cells more sensitive to radiation (Fang *et al*., 2011). Cytoglobin overexpression resulted in protection of human neuroblastoma cells against oxidative stress-induced cell death (Fordel *et al*., 2006). These studies exemplify a link between radiosensitivity and oxidative stress regulation systems that can affect the radiosensitivity of aging cells.

In summary, irradiating aging cells that already display an unbalanced oxidant–antioxidant status would unequivocally contribute to overload the antioxidant systems (Fig.1). In aging cells, when ROS production exceeds the antioxidant defense capacity of the cell, excess of oxidative stress occurs and induces damage to the DNA, proteins, and membrane lipids. In summary, during aging, radiation further contributes to ROS generation, increasing the possibilities of oncogenic transformation.

## Telomeres: effects of the age-related attrition in radiosensitivity

### Telomere attrition with age

Telomeres are DNA-protein structures at the end of the chromosomes, protecting them from exonucleolytic degradation, homologous recombination, and nonhomologous end joining. In the absence of balancing mechanisms, telomeric DNA shortens with each DNA replication. Although telomerase can add new telomeric DNA to the ends of the chromosomes, its activity is basically restricted to ovaries, testes, and highly proliferative tissues such as activated lymphocytes and adult tissue stem cells (Colgin & Reddel, 1999). Therefore, most somatic cells in human tissues have insufficient levels of telomerase activity, and telomere attrition occurs in parallel with aging in rapidly proliferating cells of the skin, gastrointestinal system, and blood. Some studies have established that the mean leukocyte telomere length is inversely associated with age and it declines between 20 and 40 bp per year (Brouilette *et al*., 2003, 2007; Fitzpatrick *et al*., 2007). Once a critical shortened telomere length is attained, it triggers a state of permanent growth arrest called replicative senescence (Harley *et al*., 1992). Replicative senescence is induced by activating a DNA damage response similar to that caused by DNA double-strand breaks (d'Adda di Fagagna *et al*., 2003) and has been postulated as a tumor suppressor mechanism. In fact, malignant progression occurs when cells acquire mutations, primarily in the p53 or Rb pathways, allowing them to overcome this telomere-dependent proliferative arrest. When aging cells bearing critically short telomeres ignore the senescence-inducing signals, or escape from the senescence growth arrest, their telomeres keep getting shorter and dangerously dysfunctional in terms of genomic instability.

### Telomere attrition and genomic instability

**Figure 2:**
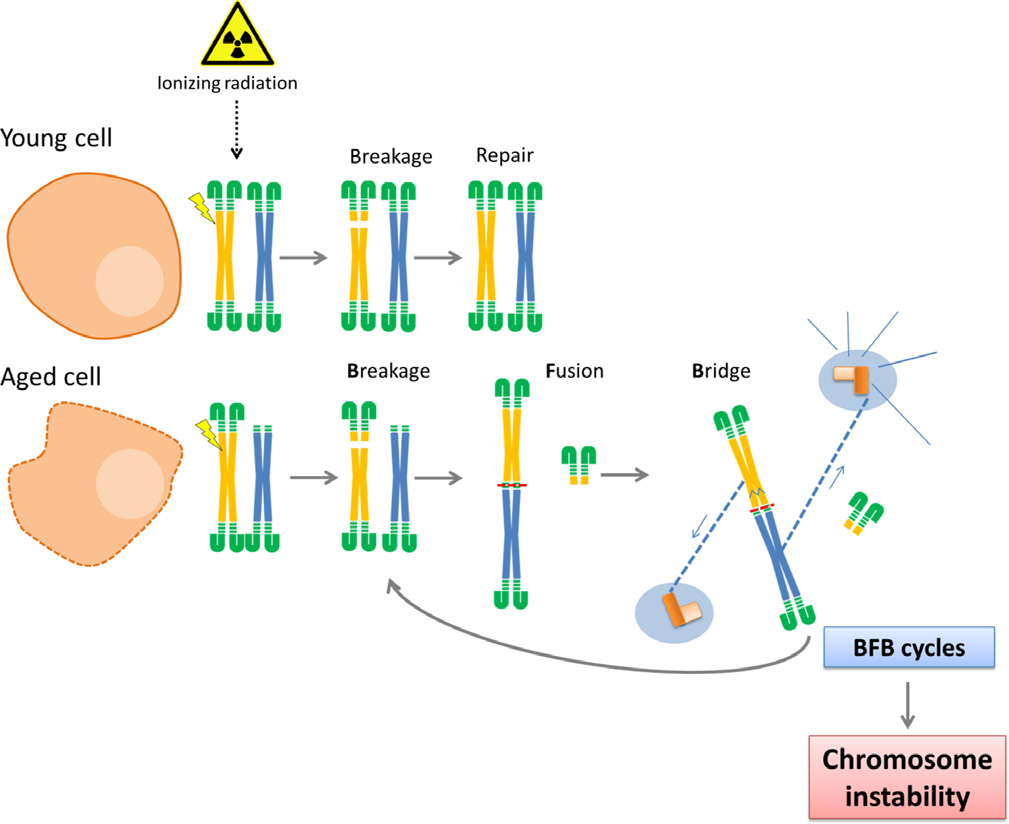
Telomere attrition in aging cells. Ionizing radiation induces new DNA double-strand breaks (DSBs), and thus, new opportunities for the uncapped chromosomes undergo unfaithful repair. When aging cells skip replicative senescence, they display a greater number of uncapped chromosomes, which are prone to produce rearrangements. They can undergo end-to-end fusions and DSB-end fusions between different chromosomes. When the two centromeres are pulled in different directions, dicentric chromosome can break, and this breakage results in further fusions followed by other bridges and, again, new breaks will arise. This process is known as the breakage–fusion–bridge cycle (BFB cycles), which leads to broad DNA amplification and progressive terminal deletions. Any of these outcomes lead to a rise in chromosome instability, which, in turn, can initiate or promote a carcinogenic process.

Cell proliferation beyond replicative senescence leads to uncapped chromosomes that can fuse with each other or with its sister chromatid after DNA replication (Soler *et al*., 2005). Such unstable chromosome configurations can set up fusion–bridge–breakage cycles, which are prone to produce rapid and important changes in gene dosage, thus linking telomere dysfunction and chromosome instability (Fig.2). Carcinogenesis is particularly induced when the cellular response to telomere attrition is reduced due to cell cycle checkpoint defects, as demonstrated by studies carried out in mice with impaired p53 function (Artandi *et al*., 2000). A dramatic increase in the incidence of tumors, specifically carcinomas, has been observed in *hTERT*^*−/−*^ *p53*^*+/−*^ mice with short telomeres. Indeed, the cytogenetic characteristics of these tumors reproduced those identified in human carcinomas, showing a high frequency of unbalanced chromosome rearrangements (Artandi *et al*., 2000). Of note, mutations and deletions of the *TP53* tumor suppressor gene encoding p53 are very frequent in human carcinomas (Negrini *et al*., 2010) and remarkably associated with organismal aging and cancer incidence (Richardson, 2013). In fact, several studies have reported an increase of DNA damage, mutations, and genome instability related to age (Burhans & Weinberger, 2007; Lushnikova *et al*., 2011; De Magalhães, 2013; López-Otín *et al*., 2013). Additionally, there might exist an age-related deficiency of the senescence response efficacy mechanisms that could lead to the proliferation of cells with critically short telomeres (Feng *et al*., 2007). And finally, the most direct evidence that telomere dysfunction is an important component of the genomic instability observed in human cancer comes from studies using a PCR-based assay to detect and analyze telomere fusions. Using a telomere-associated repeat fusion PCR, Tanaka *et al*. (2012) reported that human breast premalignant and malignant lesions, but not normal breast tissues, contained telomere fusions. Altogether, these studies support the notion that age-dependent telomere attrition in a cell environment with impaired cell cycle checkpoints contributes to human carcinogenesis in the elderly (Meeker & Hicks, 2004; Negrini *et al*., 2010).

### Telomere attrition raises radiosensitivity

Telomeric dysfunction not only underlies the initiation of carcinomas but also relates to radiosensitivity. Cells with short telomeres are more radiosensitive than their long telomere counterparts (Goytisolo *et al*., 2000; Wong *et al*., 2000). Studies in irradiated embryonic fibroblast from telomerase-deficient *Terc*^*−/−*^ mice provided an important clue to understand the basis of the increased sensitivity of this mouse model. Latre *et al*. (2003) observed that chromosomes with unprotected ends fuse, not only to one another, but also to radio-induced DNA double-strand breaks (DSBs) (Latre *et al*., 2003). Thus, shortened telomeres provide radiation-induced DSBs with a new joining possibility. If aging cells fail to trigger replicative senescence (Feng *et al*., 2007), their uncapped chromosomes will offer additional rejoining opportunities that may increase improper repair of radiation-induced breaks (Fig.2). Thus, if aging alone can induce chromosome instability when eroded telomeres meet checkpoint impairments, radiation would exacerbate this scenario by further compromising genomic stability in older organisms.

## Impaired DNA repair of the aging cells

**Figure 3:**
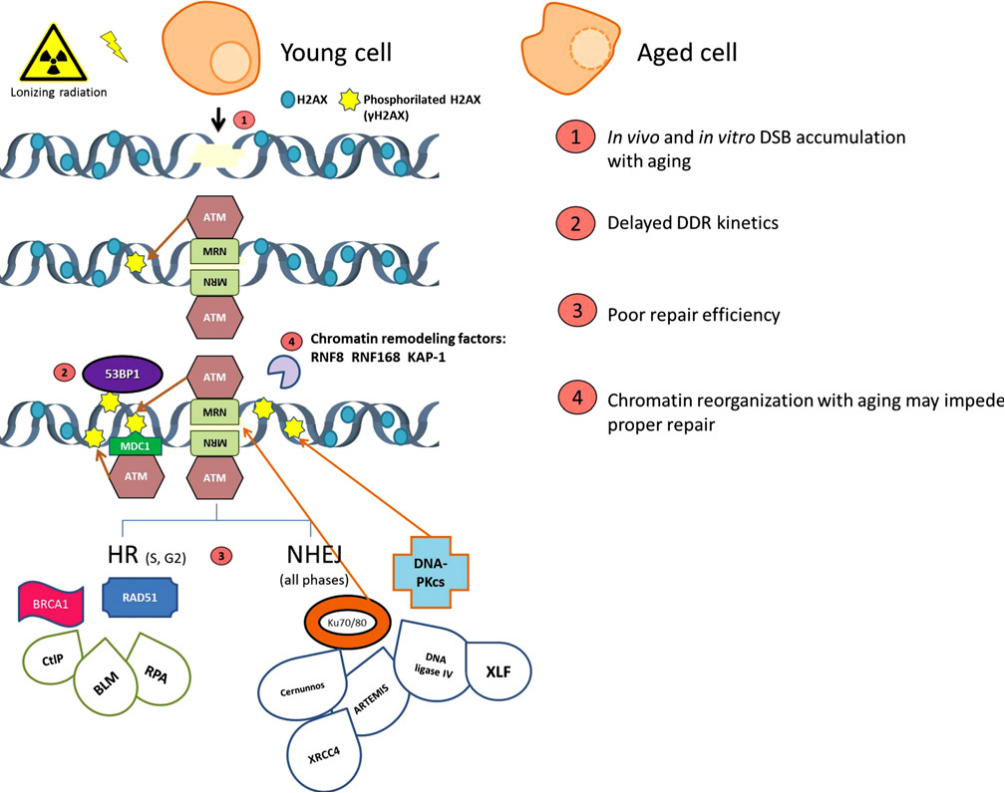
Activation of the DNA damage response (DDR) after DNA double-strand breaks (DSB) induction in young and old cells. The signaling pathway starts with the MRN complex recruitment and continues with ATM phosphorylating H2AX. As represented here, the heterodimer Ku70/80 can also interact directly with the DSB and promote H2AX phosphorylation in DSB-flanking chromatin. The modified histone form (γH2AX) triggers MDC1 and 53BP1 assembly to the DSB and subsequent binding of ubiquitin ligase RNF8 and other chromatin remodeling factors. Proteins from both the nonhomologous end joining (NHEJ) and homologous recombination (HR) pathways are also recruited at the sites of damage to repair the DSB. Aging cells show a decline in the DDR efficiency at different points of this response. These points are represented by numbers (1–4) in the scheme.

DNA is constantly under the attack of both endogenous and environmental agents. Given the importance of its preservation, cells have developed several mechanisms, commonly known as DNA damage response (DDR), for fighting against these threats. These mechanisms detect the DNA damage and mediate its repair while arresting the cell cycle to avoid DNA replication or segregation (Jackson & Bartek, 2010). When DNA damage persists unrepaired, DDR can trigger cell death by apoptosis or halt cell proliferation through induction of senescence. Among all different types of DNA lesions, DSBs are highly deleterious and their presence activates the DDR surveillance system (Bekker-Jensen & Mailand, 2010). DSBs can arise from ionizing radiation, oxidative stress, or replication stress, but can also be formed during genetically programmed processes such as meiotic recombination in germ cells and V(D)J recombination in developing lymphocytes (Wyman & Kanaar, 2006). The two main pathways responsible for DSB repair are nonhomologous end joining (NHEJ) (Lieber, 2008) and homologous recombination (HR) (San Filippo *et al*., 2008). Before repair is happening, the DSB is signaled by the DDR, resulting in the recruitment of proteins involved in any repair pathways. Phosphorylation of the histone variant H2AX (γH2AX) by ATM and DNA-PKcs represents the first event of the signaling cascade (Rogakou *et al*., 1998). γH2AX enables the binding of additional DDR factors, such as MDC1, BRCA1, and 53BP1. H2AX phosphorylation takes place at the DSB site shortly after its formation and disappears at its resolution. The phosphorylated histone H2AX is microscopically visible by immunostaining, which makes γH2AX foci a widely used surrogate to measure DSBs. We focus this part of the review on the impact of aging in proper DSB resolution (Fig.3).

### DNA damage response efficiency declines with age

It has been reported that aging cells have increased amounts of γH2AX foci as compared with younger cells, suggesting that they accumulate a higher amount of unresolved DSBs, seen as increasing γH2AX levels with age (Sedelnikova *et al*., 2008; Joyce *et al*., 2011; Rübe *et al*., 2011). Not only spontaneous foci are more abundant in these cells, but also a significant induction of damage has been observed following exposure to low doses of radiation in aging cells. In fact, we reported that X-ray doses equivalent to those applied to the breast surface after a single mammogram exploration, resulted in more γH2AX foci in the *in vitro* aged human mammary epithelial cells than in their young counterparts (Hernández *et al*., 2013). In our work, cells were transduced with hTERT, discarding that telomere attrition and its subsequent increase of misrejoining events could account for the increase in γH2AX foci. In addition, *in vitro* aged cells also showed a delay in the recruitment of the DDR protein 53BP1 to the damage site (Hernández *et al*., 2013). In line with these results, it had been previously proposed that recruitment rates of DNA repair proteins at DSB sites after irradiation might be inversely correlated with donor age (Sedelnikova *et al*., 2008). It has been reported that age may diminish the effectiveness of the two main DSB repair pathways (NHEJ and HR). Using plasmid ligation methodologies, a recent study has shown that B lymphocytes from old mice show poor NHEJ repair efficiency and increased misrepair compared with younger mice (Puthiyaveetil & Caudell, 2013). Using a similar approach, Seluanov *et al*. (2004) demonstrated that NHEJ efficiency is reduced in aging cells compared with young cells. The same authors also observed that the levels of Ku―a key component of the NHEJ pathway responsible for keeping close together the two broken DNA ends―were diminished in senescent cells (Seluanov *et al*., 2007). Reduced efficiency of NHEJ in the elderly might result in persistent DSBs leading to misrejoining events and genomic instability, and also in impaired V(D)J recombination, which could contribute to reduce the immune cell repository and thus the immune system (Puthiyaveetil & Caudell, 2013). As the immune system uses its diverse antigen receptor repertoire to prevent tumor formation and progression, NHEJ impairment could eventually lead to a carcinogenic prone scenario (Li *et al*., 2011).

Similarly to NHEJ, recent reports also suggest that the HR pathway is impaired with aging. Mao *et al*. showed that presenescent cells displayed severe difficulties in recruiting RAD51 to DNA damage sites after irradiation (Mao *et al*., 2012; Chowdhury *et al*., 2013). RAD51 is responsible for mediating the invasion of the sister chromatid to find homologous sequence partners, thus representing a key component of the HR pathway. Intriguingly, exogenous incorporation of RAD51 rescued the repair ability in middle-aged cells, but failed to do so in presenescent cells (Mao *et al*., 2012), suggesting that in the oldest cells there must be other factors responsible for the DNA repair defects. To sum up, when analyzing age-related radiosensitivity, DSB repair pathways impairment must be considered. Although the exact nature of age-related misrepair remains unknown, emerging evidence points at DNA repair proteins recruitment to the damaged DNA at the nucleus as promising targets for this yet unexplored research area.

## DNA damage, inflammation, and aging

Cells respond to foreign DNA introduced in the cytoplasm by triggering innate immune responses, which are not specific to a particular pathogen in the way adaptive immune responses are. The accumulation of bacterial or viral double-stranded DNA in endosomes and DNA by-products derived from retroviruses in the cytosol triggers immune activation (Hemmi *et al*., 2000; Ishii *et al*., 2006; Stetson & Medzhitov, 2006). Of relevance, as DNA breaks can be generated during viral integration, the nucleus is not invisible to immune DNA sensors. Consequently, nuclear DNA damage triggers a chronic autoinflammatory response (Karakasilioti *et al*., 2013). In this sense, etoposide-induced DSBs in the nuclear DNA stimulate a cascade of proinflammatory signals through the induction and action of interferon cytokines (Brzostek-Racine *et al*., 2011). The mechanism underlying this response might involve ATM, a central transducer in the DDR, because ATM-deficient cells fail to regulate interferon during the response to genotoxic stress (Pamment *et al*., 2002). However, these ATM-deficient cells can still activate interferon in response to stimuli other than DNA damage. Accordingly, ATM is not only present in the nucleus, but also in the cytoplasm (Hinz *et al*., 2010). It is possible that the DDR-dependent interferon activation has emerged as an evolutive response to DNA damage induced by viruses, working as a mechanism that reduces cell proliferation to allow DNA repair or promote cell death.

Whereas the studies mentioned so far reveal a direct link between innate immune signaling and the response of cells to induced DNA damage, aging could radically affect the scenario. Dysfunctional telomeres, DNA damage, and the persistent response to these events eventually trigger cellular senescence, a state of irreversible cell cycle arrest. Cells bearing senescent markers increase with age in a variety of tissues in mice (Krishnamurthy *et al*., 2004; Wang *et al*., 2009) and in primates (Herbig *et al*., 2006; Jeyapalan *et al*., 2007). Although senescence was initially understood as a protective mechanism to suppress the development of cancer and promote tissue repair, this cellular mechanism is now seen as a double-edged sword (Campisi, 2011). A role for senescence in tissue repair would probably explain the evolution of the so-called senescence-associated secretory phenotype (SASP) (Coppé *et al*., 2008). The SASP entails the secretion of factors, such as the proinflammatory cytokines IL (interleukin)-6 and IL-8. Although there is evidence that the SASP suppresses tumor formation by reinforcing cellular senescence, it also promotes cancer progression by stimulating the growth of nearby precancerous cells. The most convincing evidence for this activity comes from xenograft studies. Co-injection of senescent fibroblasts significantly stimulated the proliferation of mouse and human epithelial tumor cells, while co-injection of nonsenescent fibroblasts did not (Liu & Hornsby, 2007). As cancers are among the pathologies that are fueled by inflammation (Grivennikov *et al*., 2010), the cytokines that comprise the SASP in aging organisms, together with the innate immune responses triggered by DNA damage, can synergistically contribute to age-related cancer by stimulating inflammation.

## Nuclear structure and radiosensitivity

### Aging-related changes in the nuclear organization hinder proper DNA Repair

Age-related changes have been detected in the nuclear lamina and the nuclear pore complexes, the main components of the peripheral nuclear matrix. These changes can lead to chromatin reorganization and alterations in the epigenetic status. Recent studies have highlighted the relevance of the lamina in the maintenance of the nuclear architecture and genome integrity and have shown that age-related changes induce poor DNA repair efficiency and fidelity.

Lamins are type V intermediate filaments located just inside the nuclear inner membrane, forming a meshwork throughout the nucleoplasm called nuclear lamina. Two splice variants of A-type lamins, lamin A, and lamin C, together with lamin B, participate in many essential nuclear processes. Among them, lamins contribute to DNA replication and repair (Goldman *et al*., 2004; Oberdoerffer & Sinclair, 2007; Mewborn *et al*., 2010; Camps *et al*., 2014). The relationship between lamins and aging has been approached through the study of Hutchinson–Gilford progeria syndrome (HGPS), a rare condition of premature aging caused by a mutation in *LMNA*, the gene encoding A-type lamins. These mutations translate to the accumulation of an immature nonfunctional protein (progerin) that causes changes in gene expression, heterochromatin organization, and failure of proper DNA repair (Liu *et al*., 2005). Interestingly, the accumulation of the immature prelamin A has also been reported in normal dermal fibroblasts from old individuals (Scaffidi & Misteli, 2006), in *in vitro* senescent human fibroblasts (Cao *et al*., 2007), and in aged vascular smooth muscle cells (Ragnauth *et al*., 2010). Therefore, production of progerin in normal aging cells may be inducing similar phenotypic features to those reported in laminopathies such as defective DNA repair, changes in heterochromatin organization, and telomere attrition (Gonzalez-Suarez & Gonzalo, 2010).

In fact, several studies unmasked the close relationship between telomere attrition, nuclear integrity, and DNA repair. Cao and colleagues demonstrated that progressive telomere attrition in normal cells acts as an upstream signal to activate the cryptic splice site in LMNA to produce progerin. They also suggested that progerin induces an acute DNA damage response at telomeres that leads to deprotection of the telomeric 3′ overhang (Cao *et al*., 2011). In line with this, fibroblasts from LMNA null mouse model showed an altered telomere structure and function together with compromised efficiency of NHEJ and HR pathways, as recruitment of 53BP1 and RAD51 to DSBs was delayed in progeria cells (Liu *et al*., 2005; Gonzalez-Suarez *et al*., 2009). Moreover, it has been suggested that compromising the expression of lamin A causes 53BP1 degradation, as A-type lamins seem to be involved in the stabilization of 53BP1, preventing its degradation by the proteasome (Gonzalez-Suarez & Gonzalo, 2010). The loss of lamin proteins also leads to a defective HR pathway, due to the formation of p130/E2F4 complexes, which bind *RAD51* and *BRCA1* promoters and inhibit their transcription (Haithcock *et al*., 2005; Redwood *et al*., 2011). Therefore, lamins deficiency or mutation can induce unprotected telomeres and compromise the accumulation of DNA repair proteins, resulting in impaired DSB repair (Liu *et al*., 2005; Redwood *et al*., 2011). Because normal aging cells can accumulate progerin, radiation exposure and/or oxidative stress may induce genomic instability among the elderly.

Nuclear pore complexes (NPCs) can also contribute to the DDR deterioration. Dysfunction of the nuclear pore complexes can result in the loss of essential proteins to maintain the chromatin organization and DNA repair. NPCs work as nuclear gatekeepers, allowing the free diffusion of small molecules and tightly regulating the transport of macromolecules (Fernandez-Martinez & Rout, 2009). The lifespan of some scaffold nucleoporins is exceptionally long (D'Angelo *et al*., 2009), and thus, the lack of turnover contributes to NPCs deterioration by age-related oxidative stress (Savas *et al*., 2012). Importantly, damaged NPCs are more permeable, which causes leaking of cytoplasmic proteins, such as tubulin, into the nucleus (D'Angelo *et al*., 2009). Permeable NPCs could also disturb the nuclear presence of DDR factors leading to an inefficient DNA repair. Altogether, aging may compromise several roles of the nuclear envelope putting the genome integrity at risk.

### Ionizing radiation corrodes the already disturbed nuclear organization in the aging cells

**Figure 4:**
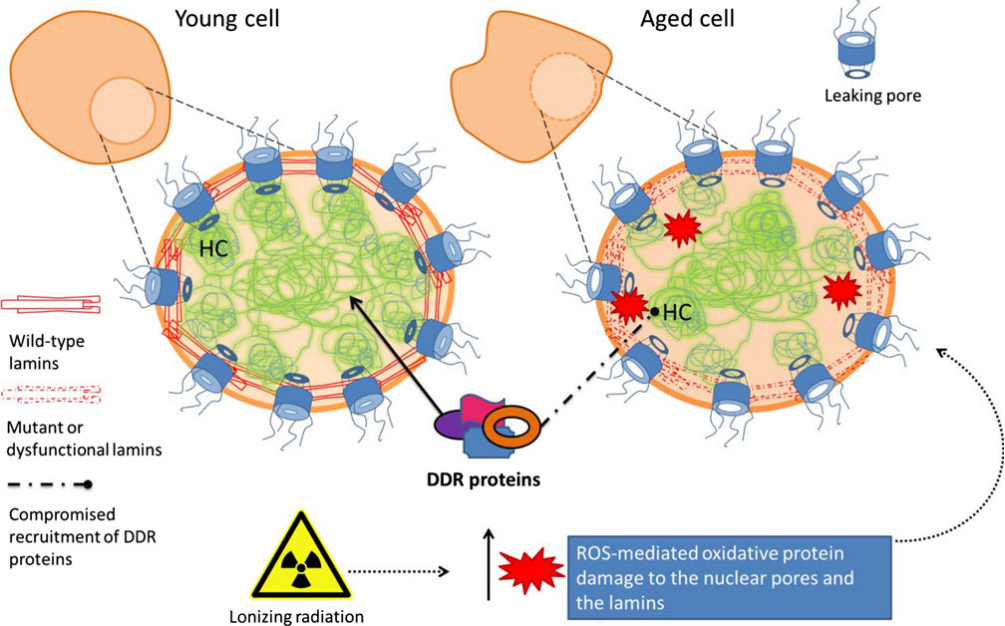
Scheme of the age-related defects in nuclear reorganization. The aging process entails changes at the nuclear organization level that may compromise the DNA damage response (DDR) proteins recruitment to the nucleus: accumulation of a premature and dysfunctional prelamin A, untethering of the heterochromatin (HC) domains, and an increased presence of leaking pores. The addition of radiation by means of reactive oxygen species (ROS) production hinders both NPC and lamins function by oxidation.

Age-related changes described above, such as nuclear lamina decay and leaking NPCs, hamper the proper repair of radiation-induced DNA lesions. Hence, the increased radiosensitivity observed in aging cells may be a consequence of already dysfunctional systems trying to cope with the damage raised by IR. As stated before, ionizing radiation does not only induce DNA lesions, but also disturbs the nuclear organization by ROS production (Fig.4). Indeed, the lamina and the NPC might be functionally modified by exposure to free radicals that accumulate in aging cells and that are derived from IR exposure. In this line, increased levels of carboxyl groups, indicative of oxidative protein damage, have been found in those nucleoporins forming age-related damaged leaky NPCs (D'Angelo *et al*., 2009). When the nuclear lamina is exposed to chronic or acute oxidative stress, irreversible damage is induced to their conserved cysteine residues, impeding their function (Pekovic *et al*., 2011; Sieprath *et al*., 2012). In light of these results, it is plausible to hypothesize that irradiating aged cells would promote further protein oxidation and, as a consequence, further nuclear disorganization hampering the repair of radiation-induced DNA lesions and thus jeopardizing the maintenance of genome integrity.

## Conclusions

There are many factors that contribute to the close relationship that exists between radiation sensitivity and age. These factors provide several plausible and nonexclusive explanations for the increased carcinogenic risk of radiation in the elderly, as reported by epidemiological studies in different cohorts. It is possible that the age-related oxidative stress accumulation disturbs the nuclear organization, which in turn may impede a proper repair. Because nuclear organization is also disturbed by ionizing radiation, aging cells become an easy target for suffering enhanced effects after irradiation. DDR impairment together with telomere attrition can result in the accumulation of genomic rearrangements that may contribute to the increased incidence of radiation-induced carcinogenesis in the elderly. Nevertheless, the intricate interplay between these aging-related defects acts as a confounding factor making it very difficult to study the contribution of each factor independently. Thus, studies that provide an integrated view of how radiosensitivity affects aging organisms are necessary for a better understanding and improvement of experimental designs. We conclude that aging must be seen as a convoluted scenario, where radiation is a bump in the road of preserving genome integrity. Because radiosensitivity increases with age and life expectancy rises steadily, there is a need for improving radiation protection measures to ensure the safety of the elderly.
